# Prebiotic Potential and Value-Added Products Derived from *Spirulina laxissima* SV001—A Step towards Healthy Living

**DOI:** 10.3390/biotech11020013

**Published:** 2022-04-26

**Authors:** Vidya Sankarapandian, Kirubakaran Nitharsan, Kavitha Parangusadoss, Prakash Gangadaran, Prasanna Ramani, Balu Alagar Venmathi Maran, Manasi P. Jogalekar

**Affiliations:** 1 Department of Microbiology, Srimad Andavan Arts and Science College (Autonomous), Affiliated to Bharathidasan University, Trichy 620005, India; vidyasankarapandian@gmail.com (V.S.); nithibscnithi@gmail.com (K.N.); balakavi2009@gamil.com (K.P.); 2 BK21 FOUR KNU Convergence Educational Program of Biomedical Sciences for Creative Future Talents, Department of Biomedical Science, School of Medicine, Kyungpook National University, Daegu 41944, Korea; prakashg@knu.ac.kr; 3 Department of Nuclear Medicine, School of Medicine, Kyungpook National University, Daegu 41944, Korea; 4 Dhanvanthri Laboratory, Department of Sciences, Amrita School of Engineering, Amrita Vishwa Vidyapeetham, Coimbatore 641112, India; r_prasanna1@cb.amrita.edu; 5 Center of Excellence in Advanced Materials & Green Technologies (CoE–AMGT), Amrita School of Engineering, Amrita Vishwa Vidyapeetham, Coimbatore 641112, India; 6 Borneo Marine Research Institute, Universiti Malaysia Sabah, Kota Kinabalu 88400, Sabah, Malaysia; 7 UCSF Helen Diller Family Comprehensive Cancer Center, San Francisco, CA 94158, USA

**Keywords:** cyanobacteria, *Spirulina laxissima*, biomass, chlorophyll, phycocyanin, value-added products

## Abstract

Lately, microalgae-based value-added products have been gaining market value because they moderate the dependency on fossil fuel and high-value chemical products. To this end, the purpose of this study was to develop prebiotic products from the microalgae *Spirulina* sp. The microalgae were isolated from the fresh water and characterized at the molecular level. The dry biomass, chlorophyll content, phycocyanin, cytotoxicity and antimicrobial and antioxidant properties of the isolated strains were analyzed. Moreover, value-added products like *Spirulina* cake, chocolate, tea, vermicelli and *Spirulina* juice were made for a vulnerable population due to high nutritive value.

## 1. Introduction

Malnutrition is a worldwide public health problem [1]. The Food and Agriculture Organization (FAO) of the United Nations estimates that 2 billion people suffer from malnutrition globally [2], World Health Organization (WHO) defines malnutrition as “inadequate or excess intake of protein, energy and micronutrients such as vitamins, and frequent infections and disorders” [3]. Many researchers are working to overcome these concerns and develop a cost-effective product. *Spirulina* is one such source that is packed with a wide range of macro- and micronutrients benefitting human health, and is easy to produce, process and distribute [4]. As one of the best fast-growing cyanobacteria, the *Spirulina* species on earth has great potential to satisfy the ever-increasing demand in food, energy and material in a sustainable way [5]. They are photosynthetic microorganisms that utilize photosynthetic active radiation (PAR), carbon dioxide (CO_2_) and nutrients to grow rapidly while floating on water [6]. They are 250 µm in size. The growth conditions for their cultivation and biomass production include adequate light (2300 Lux), temperature (33 °C), CO_2_, nitrogen, phosphorus and trace elements [7]. *Spirulina* grows in subtropical and tropical lakes that have high levels of the minerals, especially carbonate and bicarbonate [8]. It seems to have a positive effect on immunity and has several health benefits [9].

In the present study, we have isolated, identified and formulated various products that are useful in daily life such as soap, vermicelli, capsules, tea, chocolate, etc. Addition to the products the biomass, chlorophyll, phycocyanin, antimicrobial and antioxidant content of *Spirulina laxissima* SV001 was also determined to check the prebiotic potentials of the isolated strains.

## 2. Materials and Methods

### 2.1. Study Area, Sample Collection and Isolation of Spirulina sp.

The algal samples were collected from the Gundur lake, Tiruchirappalli District, Tamil Nadu, South India with exact Latitude −10°43′ N and Longitude −78°43′ E in the central Tamil Nadu. The samples collected were screened for the *Spirulina* species. Fresh cyanobacterial species were collected in 250 mL plastic bags from the upper part of the lake. The collected algal cultures were transported to the laboratory. The collected samples contain different kinds of microorganisms in addition to some abiotic components. The contaminants were removed using density gradient centrifugation. Density gradient is one of the most rapid and sensitive techniques for algal separation [10]. Repeated low-speed centrifugation (500–1000 rpm) removed up to 90% of the suspended materials and unicellular microorganisms. *Spirulina* was found at the upper phase in the floating mass. The *Spirulina* was transferred to BG11 media for cultivation [11].

### 2.2. Medium for Maintaining and Harvesting Spirulina sp.

The freshwater cyanobacterial cultures used in the study were grown photoautotrophically under continuous illumination with white fluorescent light at ~200 μmol photon m^−2^ s^−1^ in Erlenmeyer flasks containing BG11 medium at 35 ± 2 °C for a week [11,12]. After the incubation time of 7–10 days, the cultures were sieved by the cotton cloth of porosity 100–150 μm [12]. The excess water from the slurry was removed. The slurry was allowed to dry on a plain cloth and stored for future use.

### 2.3. Morphological Studies

Pure colonies were observed under the microscope for taxonomic identification. The *Spirulina* species were identified taxonomically according to Ciferri, (1983) [13] and Tomaselli, (1997) [14].

### 2.4. Molecular Identification of the Isolated Strain Using 16S rRNA

Bacterial DNA extraction, PCR amplification, and DNA sequencing of the 16S rRNA gene were performed [15].

### 2.5. DNA Extraction

The algal cells were grinded with the help of mortar and pestle to remove phenolic contamination. The adequate amount of cetyl trimethylammonium bromide (CTAB) was added to cells to make fine slurry and 1.5 mL of slurry was transferred to the 2 mL eppendorf tube. 10 μL of β-mercaptoethanol was added and the mixture was incubated at 65 °C for 1 h in the water bath. The supernatant was collected following the centrifugation at 13,000 rpm for 15 min. Equal amount of chloroform:isoamyl alcohol (24:1) was added to the supernatant. The top layer was collected in a fresh eppendorf tube, and an equal volume of chilled isopropanol was added. The DNA was then precipitated by incubating for 20 min at 20 °C and centrifuging at 13,000 rpm for 15 min. The supernatant was discarded, and the pellet was washed with 500 μL of 70% ethanol. The solution was centrifuged at 8000 rpm for 5 min. The supernatant was discarded and 70 μL of TE buffer was added. DNA concentrations were measured by agarose gel (1%) electrophoresis. The samples were stored at −20 °C until further use.

### 2.6. Primer Design and Polymerase Chain Reaction

Polymerase chain reaction (PCR) uses the enzyme DNA polymerase that directs the synthesis of DNA from deoxynucleotide substrates on a single-stranded DNA template. DNA polymerase adds nucleotides to the 3’end of a custom-designed oligonucleotide when it is annealed to a longer template DNA. Thus, if a synthetic oligonucleotide is annealed to a single-stranded template that contains a region complementary to the oligonucleotide, DNA polymerase can use the oligonucleotide as a primer and elongate its 3’ end to generate an extended region of double-stranded DNA.

The forward and the reverse primers were 27F contains 20 base pairs (5′ AGAGTTTGATCMTGGCTCAG 3′) and 1492R contains 20 base pairs (5′ TACGGYTACCTTGTTACGACTT 3′), respectively. The PCR reaction solution (1.5 μL each of Forward and Reverse Primer, 5 μL of deionized water, and 12 μL of Taq Master Mix) was added to 5 μL of isolated DNA for the total of 25 μL solution. The PCR was performed in 25 µL reaction volumes containing 1X Taq Master Mix (Merck, Bangalore, India), 1.5 mM MgCl_2_ (Merck), 0.25 mM forward primer, 0.25 mM reverse primer and 0.4 ng of genomic DNA. Temperature cycling for PCR started with the initial denaturation step at 95 °C for 3 min, followed by 30 cycles, each at 95 °C for 50 s. Annealing temperature was 58 °C for 50 s and 72 °C for 1 min, with a final extension at 72 °C for 7 min. The quantity of PCR reaction was monitored in 1% Trisacetate- EDTA- agarose gel (Sigma Aldrich, Bangalore, India) and bands were visualized by staining with ethidium bromide (Sigma Aldrich, Bangalore, India). Sequence of 16S rRNA gene was generated from for-ward and reverse sequence data using aligner software and sequenced in Applied Bio-Systems (Bangalore, India). The sequences generated from the isolated strains were submitted in NCBI.

### 2.7. Growth Parameters

#### 2.7.1. Determination of Dry Biomass

Biomass concentration was calculated by measuring dry weight. For dry weight measurement, homogenous suspensions of known quantity of the *Spirulina* sample were filtered through the screen-printing paper and oven-dried at 75 °C for 4 to 6 h [16]. The dried filter paper containing *Spirulina* biomass was cooled and weighed. The difference between the initial and final weight was taken as the dry weight of *Spirulina* biomass.

#### 2.7.2. Chlorophyll Estimation

Methanol of about 80% was prepared by mixing 80 mL of methanol and 20 mL of distilled water. The cultures were centrifuged at 10,000× *g* for 10 min. The water-washed pellet was suspended in 5 mL of 80% methanol and incubated at 60 °C for 1 h [17]. The tubes were then centrifuged at 5000× *g* for 5 min. and the supernatant was examined. The absorbance was measured at 663 nm. The amount of chlorophyll a was calculated as follows:

Chlorophyll a (µgmL^−1^) = A663 × 12.63 × volume of methanol

#### 2.7.3. Extraction of Phycocyanin

The phycocyanin was extracted from *Spirulina* using freeze-thawing method [18]. Harvested biomass was homogenized using a hand homogenizer for 20 min. in the presence of phosphate buffer (pH 6.8) in the ratio of 1:3. The homogenized culture was freeze-thawed. The samples were then centrifuged at 3000 rpm for 45 min. The supernatant was transferred to sterile plastic tubes, covered with aluminum foil to avoid the penetration of light and stored at 40 °C for further analysis. The crude phycocyanin concentration was calculated spectrophotometrically by measuring the absorbance at 615 nm and 652 nm using the following calculation.

PC = OD _615_ − 0.474 × OD _652/_5.34, whereas 5.34 = constant

#### 2.7.4. Estimation of Isolated Protein by Lowry’s Method

The protein from *Spirulina* cultures was quantified using Lowry’s method, with bovine serum albumin as the standard [19].

### 2.8. Antimicrobial and Antioxidant Activity of the Isolated Strain

#### 2.8.1. Preparation of Aqueous Extract of *Spirulina*

The *Spirulina* powder (20 g) was soaked in 1 L of ultrapure water and shaken continuously for 24 h at room temperature. The mixture was then centrifuged at 5000 rpm for 10 min (4 °C) and the supernatant was filtered (using Whatman No. 1 filter paper) to remove the cell debris. The sample was then freeze-dried and the dried extract was stored at 4 °C until further use [20].

#### 2.8.2. Antibacterial Activity

The antimicrobial activity determination was performed using gram positive bacterium Staphylococcus aureus and five-gram negative bacteria such as *Escherichia coli*, *Klebsiella pneumoniae*, *Serratia marcescens*, *Proteus vulgaris* and *Pseudomonas aeruginosa*. The strains were collected from K.A.P. Viswanathan Medical College. They were subcultured in their selective media. Disc diffusion method [21] was followed to determine the antibacterial activity of the isolated strain. Log phase petriplates containing 20 mL of Muller Hinton agar (HiMedia) were seeded with 4 h old fresh culture of pathogens. The aqueous extract of 10, 20, 30 and 40 µL was loaded on the discs, which were then dispensed on the solidified Muller Hinton agar with test organisms and incubated at 37 °C for 24 h in an incubator. The zone of inhibition was measured using an antibiotic zone scale (HiMedia). Ampicillin was used as the positive control. Most of the studies revealed that minimum inhibitory concentration of ampicillin ranges between 1.0 and 32 µg/for various organisms. Hence, 30 µg of ampicillin was used as a positive control [22,23]. 

#### 2.8.3. Antioxidant Activity of *Spirulina*

The antioxidant activity of *Spirulina* water extracts was determined by 1, 1-diphenyl 2-picrylhyorazyl (DPPH) radical scavenging assay. The DPPH free radical scavenging activity of *S. platensis* water extract was determined by following the method of Choi et al. (2000) [24]. Butylated hydroxytoluene (BHT) was used as a reference. The radical scavenging activity of water extracts of isolated strains (0.5, 1.0 and 1.5 g/100 mL) was expressed as (%) inhibition of DPPH. The inhibition percentage was calculated according to the following equation of Yen and Duh (1994) [25].

Ip = A_B_ − A_A_/A_B_ × 100

where:

Ip: Inhibition percentage

A_B_: Absorbance values of the blank samples checked after 70 min

A_A_: Absorbance values of S. platensis solutions checked after 70 min

#### 2.8.4. Cytotoxic Activity of *Spirulina*

Cytotoxicity of the isolated strain was determined against brine shrimp [26]. One mL of water extract of the *Spirulina* strain was inoculated in 10 mL of seawater containing 10 nauplli. The experiment was performed in replicates. The above mixture without extract was used as a control. Survivors were counted at an interval of 24 h using a dissection microscope (Olympus, Tokyo, Japan), and the percentage of mortality was calculated for the experimental and control groups. Potassium dichromate served as the positive control for this brine shrimp lethality assay.

### 2.9. Value-Added Products of Spirulina

#### 2.9.1. *Spirulina* Capsule

The dried *Spirulina* was grinded smoothly and 500 mg of the powder was poured into the capsule covers and sealed. 

#### 2.9.2. *Spirulina* Soap

One liter of coconut oil was steam-boiled at 80 °C for 15 min. Sixty grams of sodium hydroxide were mixed with 140 mL distilled water in a beaker. Both the solutions were mixed homogeneously. Three grams of powdered *Spirulina* and a gram of lime peel were added to the mixture. The mixture was poured into the soap molds and allowed to solidify for one week. The pH of the soap was noted.

#### 2.9.3. *Spirulina* Chocolate

235 mL of water was boiled in a pot. 220 g of cocoa powder and 170 g of butter (creamed) was added to the pot, followed by 30 g of powdered sugar, 150 mL of milk and 500 mg of *Spirulina* powder. The mixture was then stirred until it became smooth and poured into a mold. The chocolate was allowed to harden and then popped out.

#### 2.9.4. *Spirulina* Juice

The lime was cut, and the lemon juice was extracted. It was then added to 500 mL of water, along with 3 teaspoons of sugar and 500 mg of *Spirulina*.

#### 2.9.5. *Spirulina* Tea

A mixture of dried lime peel and 500 mg of *Spirulina* powder was added to the empty tea bag and sealed. The tea bag was dipped into the boiled milk containing jaggery and mixed well. 

#### 2.9.6. *Spirulina* Vermicelli

A mixture of 1 kg of flour, 1 g of butter and 1 g of *Spirulina* was prepared. A smooth dough was made by adding sufficient water to the mixture. The dough was pressed by the squeezer to get vermicelli, which was then dried under sunlight.

#### 2.9.7. *Spirulina* Cake

One kg of flour, 1 g of baking powder, and 0.5 g of baking soda were mixed with condensed milk, melted butter, vanilla essence, 50 mg of *Spirulina* and water. The mixture was poured into a greased tin and baked in a hot oven at 200 °C (400 °F) for 10 min. The temperature was then reduced to 150 °C (300 °F) and the cake was baked for another 10 min. The cake was taken out of the oven when it left the sides of the tin and was springy to touch, then allowed to cool for a minute. The tin was inverted over a rack and tapped sharply to remove the cake. 

### 2.10. Statistical Analysis

The mean and standard deviation were evaluated based on the data obtained in triplicate. All the data were analyzed using Student’s *t*-test, and statistically significant results were examined at *p*-value < 0.05.

## 3. Results and Discussion

### 3.1. Study Area, Sample Collection and Cultivation of the Isolated Strain

**Figure 1:**
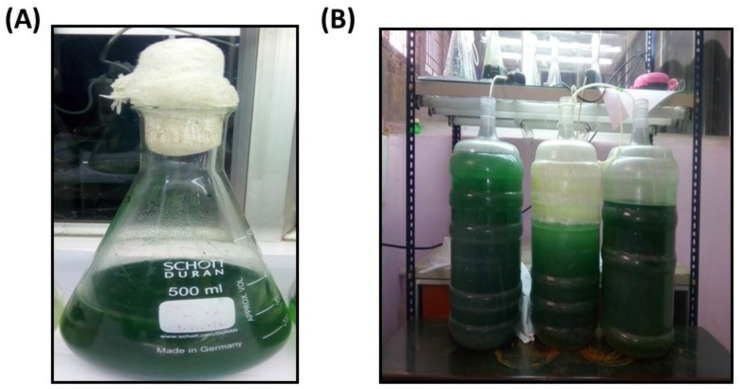
Cultivation of *Spirulina* and its scale up process. (**A**) Small scale cultivation of *Spirulina* in conical flask (**B**) Mass cultivation of *Spirulina* sp.

Since *Spirulina* is a filamentous blue-green alga, it has been collected from the upper part of the freshwater lake, Trichy. Moreover, *Spirulina* inhabit the widest variety of freshwater habitats on earth and can become important in surface blooms in nutrient-rich standing waters [27]. The collected samples were immediately sent to the laboratory and transferred to the BG11 medium since it is a freshwater algae [11] Initially, the algae were cultivated in a small quantity in a 250 mL flask, and the culture was later scaled up to 5 L (Figure 1). 

### 3.2. Morphological and Molecular Identification

**Figure 2:**
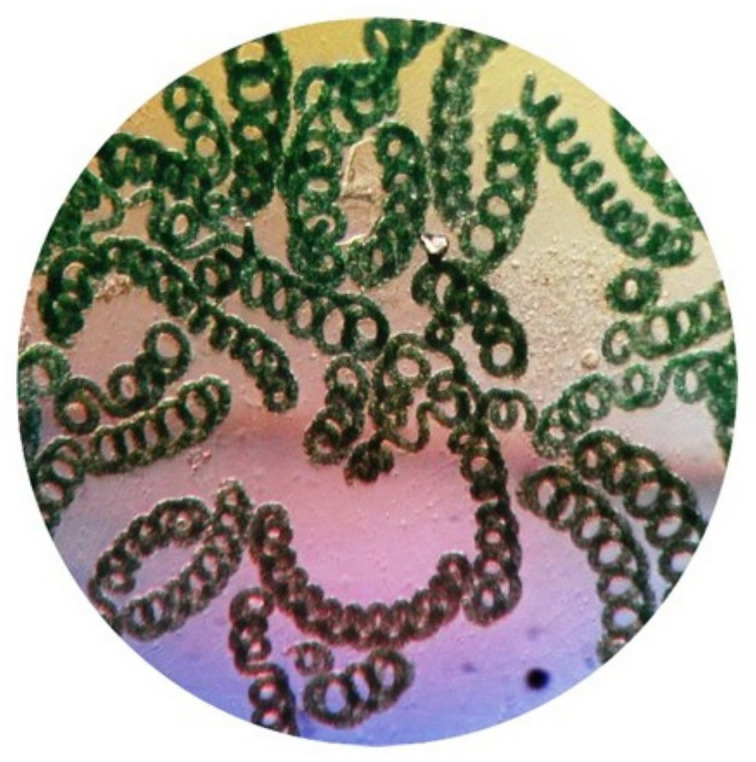
Shows the morphological structure of *Spirulina laxissima* SV001 under light microscope (40× magnification).

**Figure 3:**
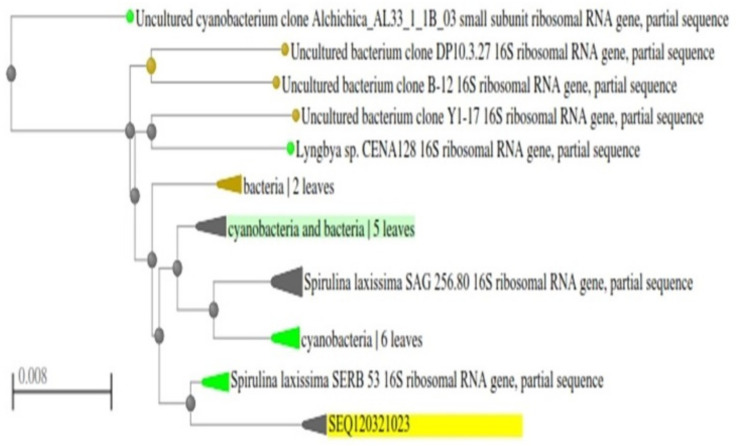
Shows the phylogenetic analysis of the isolated strain. The phylogenetic tree reveals the isolated strains belonging to *Spirulina laxissima* (NCBI Accession number: MW829712.1). The blast similarity is 99.40% with *Spirulina laxissima* SERB 53.

The morphology of the isolated algae was analyzed using a light microscope at 40× magnification (Figure 2). The *Spirulina* is characterized by its regularly coiled trichrome and is green in color. The partial sequence of 16S ribosomal RNA (rRNA) gene revealed that the rRNA is 1331 bp, and the phylogenetic tree (Figure 3) analysis showed that the strain is *Spirulina laxissima*, which is mostly identified as a freshwater algae [28]. The phylogenetic tree also reveals that the blast similarity is 99.40% with *Spirulina laxissima* SERB 53. The identified strain was named *Spirulina laxissima* SV001, and the sequence was submitted to GenBank (Accession number: MW829712.1). 

### 3.3. Biomass and Chlorophyll Content of Isolated Spirulina

**Figure 4:**
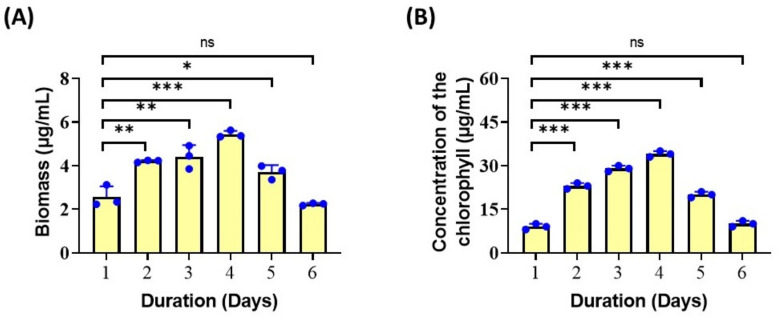
Estimation of dry biomass and chlorophyll contents. (**A**) The measurement of dry biomass of the isolated strains at day 1 to day 6, *(n* = 3). (**B**) The measurement of chlorophyll contents from day 1 to day 6, (n = 3). * *p* < 0.05; ** *p*< 0.01; *** *p* < 0.001 and ns: not significant. The student’s *t*-test was used for comparison.

The Biomass and chlorophyll content of *Spirulina* was calculated for six days by the method of MacKinney [17]. (Figure 4). The chlorophyll content gradually increases as the biomass of the *Spirulina* increases. The optimum growth was obtained during the 3rd and 4th day of the cultivation, and it was calculated as 4.92 µg/mL and 5.62 µg/mL, respectively. The biomass and chlorophyll content were found to increase for the first four days of cultivation and decrease during the days following. This was mainly due to the depletion of nutrients in the culture medium. The biomass and the chlorophyll content gradually increased following the replenishment of nutrients in the media. Thus, the alterations in the growth pattern of the *Spirulina* were observed depending upon the nutritional content of the culture medium [29]. Several reports suggest that the biomass and chlorophyll content also depend upon the temperature and pH [30,31,32]. The studies of Michaelet al. (2019), reveal that the biomass content of the algae also depend upon the type of media used for cultivation [33]. In our study, BG 11 media was used for the cultivation of *Spirulina,* and BG 11 media was evaluated as a suitable media for the cultivation of *Spirulina* [34]. Regarding the chlorophyll concentration, the studies of Gitelson, et al. (1995), states that chlorophyll biosynthesis of *Spirulina* can be favored by the composition of the cultivation medium and by the illumination [35]. In the current study, 6.0 k lux of light intensity was supplied to achieve optimum growth of *Spirulina*.

### 3.4. Phycocyanin Concentration

**Figure 5:**
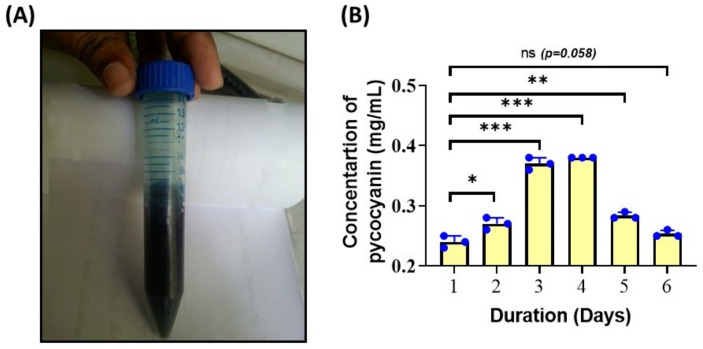
Extraction of phycocyanin and determination of its concentration (**A**) Picture shows the homogenized and centrifuged freeze-thawed culture of *Spirulina* sp. (**B**) Graph shows the concentration of Phycocyanin at different days of cultivation of *Spirulina* sp. * *p* < 0.05; ** *p* < 0.01; *** *p* < 0.001 and ns: not significant. The student’s *t*-test was used for comparison.

Biopigments are gaining importance because they are used as a natural colorant in food, cosmetics, pharmaceutical products and have tremendous applications in nutraceuticals, therapeutics and biotechnological research [36]. The studies of Tan et al. (2020), state that the freezing-thawing method is the best method for phycobiliprotein extraction technique [37]. Hence, the Phycocyanin content was analyzed using the freeze-thawing method since the release of phycocyanin is related to the cell rupture. The *Spirulina* has resistant, multi-layered cell walls, making the extraction procedure difficult [38]. The highest concentration of phycocyanin-0.36 and 0.38 mg/mL-was noted on days 3 and 4 of the cultivation, respectively (Figure 5). The results are in accordance with those obtained for the biomass and chlorophyll content. Previous reports of Morançais et al. (2018)**,** indicate that C-phycocyanins (C-PC) isolated from *S. platensis* have therapeutic benefits such as free radical scavenging activity, and antioxidant, anti-inflammatory and anti-cancer properties [39]. Therefore, it is critical to analyze phycobiliproteins from *Spirulina* sp. 

### 3.5. Protein Estimation by the Lowry Method

**Figure 6:**
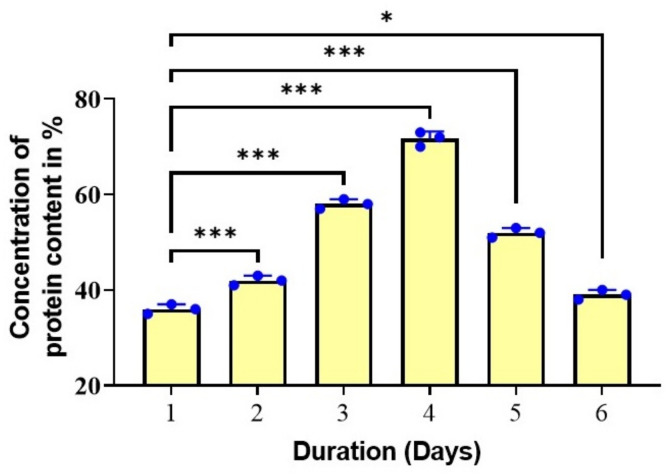
Estimation of protein content of the isolated *Spirulina* sp. The graph shows the concentration of protein of the isolated strains at different days of cultivation of *Spirulina* sp. * *p* < 0.05 and. *** *p* < 0.001 The student’s *t*-test was used for comparison.

*Spirulina* is about a sixty percent complete, highly digestible protein; it contains all essential amino acids [40]. Hence *Spirulina* is considered as Spirulina-food of the past, present and future [41]. Hence the protein content of the isolated *Spirulina* sp. was determined by the Lowry assay. The protein content in the initial day was 36% and it was 72% and 39% on day 4 and 6, respectively (Figure 6) The protein content of the *Spirulina* increased as the biomass increased, and vice versa, suggesting that the biomass and the protein content are directly proportional to each other. These findings are consistent with previous reports [42]. The studies of McCarty, (2007) showed that, C-phycocyanin contains phycocyanobilin, and it is the major protein present in Spirulina and accounts for about 20% of algae’s dry weight [43]. Soni et al. (2012) reported the production of 55% and 56.5% protein in the laboratory and outdoor environment, respectively [44]. Moreover, owing to its nutrient content *Spirulina* products are also formulated for the benefits for reducing body fat, waist circumference and body mass index [45]. 

### 3.6. Antibacterial Activity of Spirulina sp.

**Figure 7:**
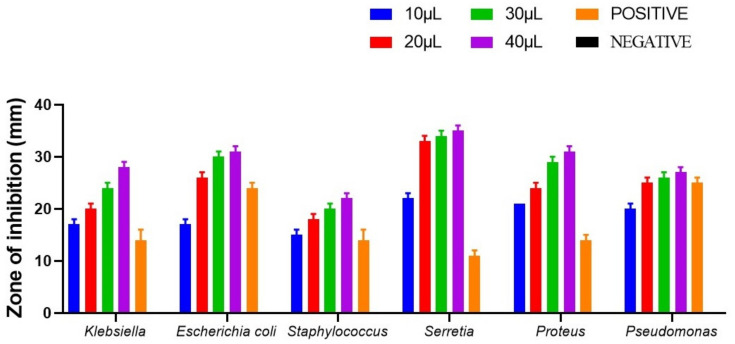
Graphical representation of the antimicrobial activity of the aqueous extract of *Spirulina laxissima* SV001 against different bacterial pathogens. *p* < 0.001, the two-way ANOVA was used for comparison.

**Figure 8:**
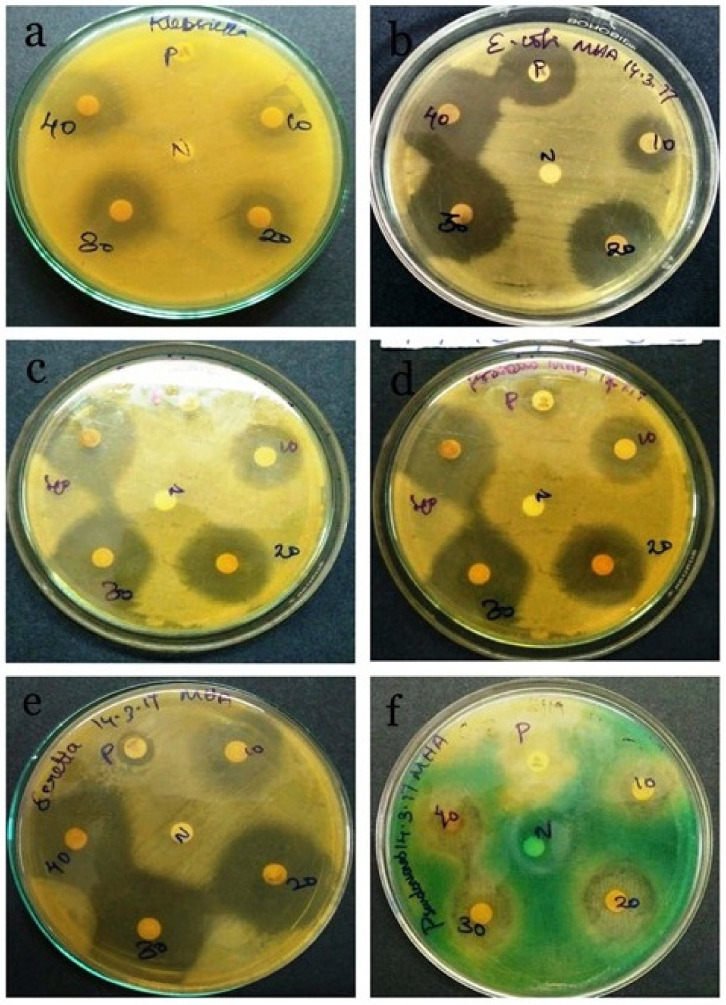
The plates represent the antimicrobial activity of the aqueous extract of *Spirulina* *laxissima* against different bacterial pathogens. The zone of inhibition of *Spirulina* extract against (**a**) *Klebsiella pneumoniae* (**b**) *Escherichia coli (***c**) *Staphylococcus aureus* (**d**) *Proteus vulgaris* (**e**) *Serratia marcescens* (**f**) *Pseudomonas aeruginosa*.

*Staphylococcus aureus*, *Escherichia coli*, *Klebsiella pneumoniae*, *Serratia marcescens*, *Proteus vulgaris* and *Pseudomonas aeruginosa* were used to check the antimicrobial activity of the isolated strain of *Spirulina* (Figure 7 and Figure 8). In our current analysis both the gram positive and gram-negative bacteria were taken for the analysis to check the wide spectrum activity of *Spirulina* extract. While all the strains exhibited varying degrees of antimicrobial activity, Spirulina exhibited the maximum antimicrobial activity against *S. marcescens* (36 mm ± 0.12), followed by others including *E. coli* (31 mm ± 0.10), *P. vulgaris* (30 mm ± 0.31), *K. pneumoniae* (27 mm ± 0.12) and *P. aeruginosa* (26 mm ± 0.10). The minimum activity was observed for *S. aureus* (21 ± 0.11). Ampicillin was used as the positive control, since Ampicillin is a β-lactam/β-lactamase inhibitor combination with a broad spectrum of antibacterial activity against, Gram-negative, Gram-positive and anaerobic bacteria [46]. Likewise, as with any other beta-lactam antibiotics, the mode of action of ampicillin is to target the primary receptors called membrane-bound penicillin-binding proteins, and these proteins perform vital roles in cell cycle-related, the morphogenetic formation of cell wall peptidoglycan [47]. Hence, it is evident that the ampicillin works on both gram positive and gram-negative organisms. Regarding the antimicrobial activity, our findings are in accordance with a previous report by Sudha et al. (2011), where the authors demonstrated that the antimicrobial activity of Spirulina is mainly due to the presence of primary and secondary metabolites [48]. Elshouny et al. (2021), isolated the antibacterial substance responsible for the antimicrobial activity of spirulina, and it was later identified as an aliphatic compound having different active groups (-OH,-C=O,-CH_2_ and –CH_3_) [49]. A previous study revealed that the hexane extract of Spirulina platensis produces minimum inhibition zone against bacterial and fungal pathogens when compared with other solvent extracts [50]. We are the first ones to report the antimicrobial activity of *Spirulina laxissima*. 

### 3.7. Antioxidant Activity of Spirulina

**Figure 9:**
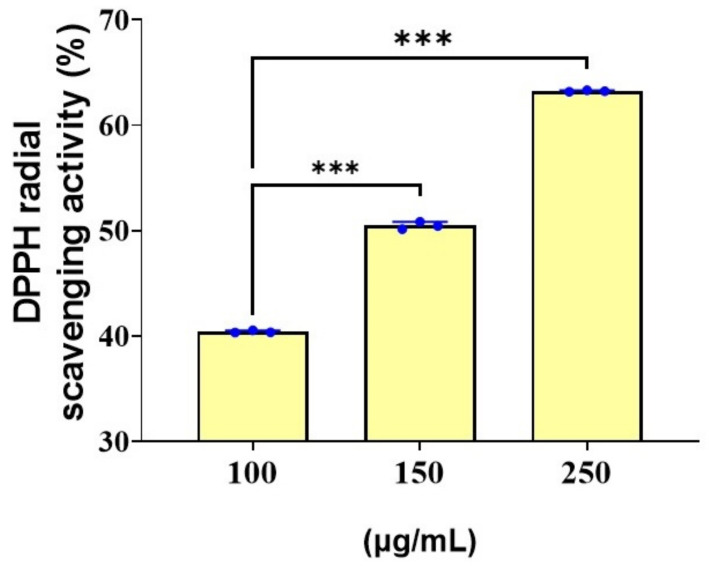
The figure shows the DPPH antioxidant activity of the isolated *Spirulina* sp. *** *p* < 0.001. The student’s *t*-test was used for comparison.

In our investigation, the highest and the lowest scavenging activity of *Spirulina* was observed at 250 mg/100 mL and 100 mg/100 mL, respectively (Figure 9). A similar study stated that the antioxidant activity of the ethanol extracts of *Spirulina laxissima* might be attributable to its proton-donating ability [51]. Miranda et al. (1998), reported the presence of phenolic acids, tocopherols and beta-carotene in Spirulina, all of which are known to exhibit antioxidant activity [52]. Another study suggested that the increase in the phycocyanin content was related to an increase in the antioxidant activity in different fractions, which supports our findings [53]. Bashandy et al. (2016), found that the antioxidant potential of *Spirulina platensis* ameliorates the arsenic-induced oxidative stress in male rats [54].

### 3.8. Cytotoxic Activity of Spirulina

Brine shrimp lethality bioassay is a simple, high throughput cytotoxicity test of bioactive chemicals. It is based on the killing ability of test compounds on a simple zoological organism-brine shrimp (*Artemia salina*) [55]. It is a convenient system for monitoring biological activities. Although this method does not provide adequate information regarding the mechanism of toxic action, it is a very useful method for the assessment of the toxic potential of various extracts [56,57]. This method provides preliminary screening data that can be backed up by more specific bioassays once the active compounds have been isolated [58]. The toxicity of the isolated water extract (1000 µL) was tested. Most experiments that involve the brine shrimp lethality assay for toxicity assessment of extracts include a concentration range of 10, 100 and 1000 µg/mL [59]. In the current investigation, the survival number of nauplii was determined at 96 h. In control, 9 nauplii survived. Eighty percent of the nauplii treated with the water extract of the *Spirulina* were survived. The cytotoxicity was considered significant if the LC50 value was less than 5 nauplii per 10 mL water [26]. Potassium dichromate served as the positive control for this brine shrimp lethality assay. No naupili survived after 12 h when exposed to positive control. Moreover, the studies of Sahgal et al. (2010), confirmed that the brine shrimp lethality assay was useful for the screening of the plant extract, to predict the toxicity level [60].

### 3.9. Value-Added Products of Spirulina

**Figure 10:**
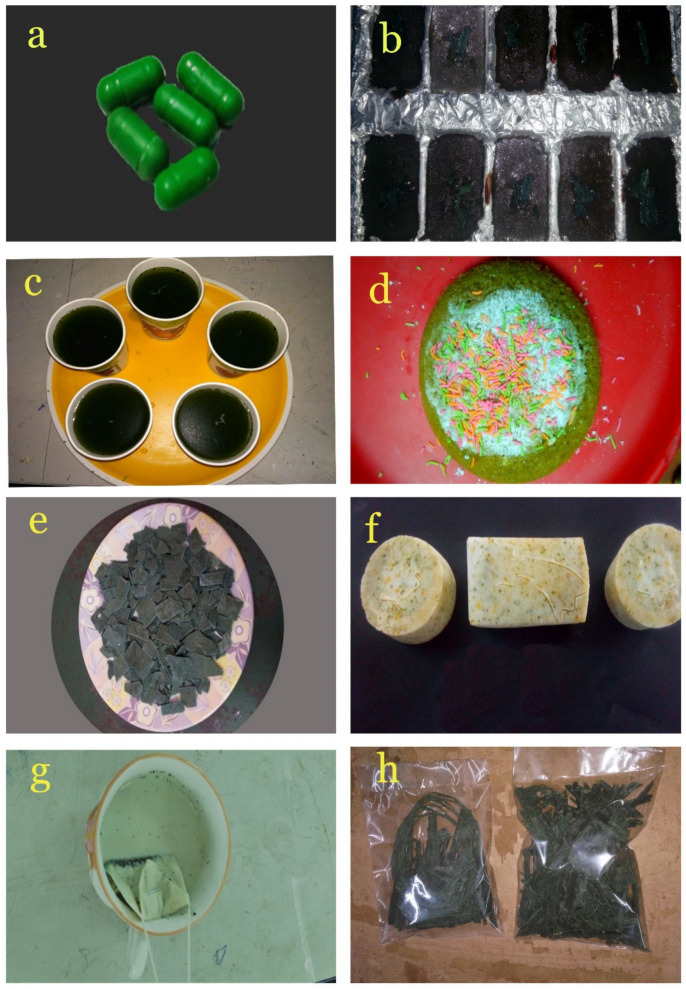
Value-added products from *Spirulina* sp. (**a**) *Spirulina* Capsule, (**b**) *Spirulina* Chocolate, (**c**) *Spirulina* Juice, (**d**) *Spirulina* Cake, (**e**) *Spirulina* Pasta, (**f**) *Spirulina* Soap, (**g**) *Spirulina* Tea, (**h**) *Spirulina* Vermicelli.

Our findings demonstrate that the *Spirulina* is a cyanobacterium with an excellent protein, biomass, chlorophyll and phycocyanin content. It also exhibits antibacterial and antioxidant activity. Therefore, we decided to use *Spirulina* to make value-added products such as *Spirulina* Capsule, *Spirulina* Chocolate, *Spirulina* Juice, *Spirulina* cake, *Spirulina* Pasta, *Spirulina* soap, *Spirulina* Tea and *Spirulina* Vermicelli (Figure 10). While the recommended dosage of *Spirulina* varies according to need, less than 1 g of *Spirulina* was added to make these products. Since *Spirulina* is very dry and dense in nature, it was well ground or pulverized to make it easier to dissolve in cold liquids and mostly mixed with warm foods to help retain the nutritive value of the powder as opposed to those foods that are at high temperature (not over 37 °C). Moreover, the high protein content, chlorophyll content, antioxidant property and antimicrobial property make *Spirulina* a potent prebiotic. Nowadays, micro-algal-based value-added products are gaining a huge market value since they moderate the dependency on fossil fuel and high-value chemical products. Therefore, our study focused on developing prebiotic products from the microalgae *Spirulina* sp. has important future implications. It should be noted that the products that developed in our study are only models and for research purposes only. They are not used for any commercial purpose or human consumption. Further, these products will be sent to (FSSAI) Food Safety and Standards Authority of India for the biosafety analysis and approval for human consumption. 

## 4. Conclusions

The purpose of the current study was to isolate *Spirulina* sp. and to unleash its prebiotic potentials. In this study, freshwater *Spirulina* has been isolated and identified as *Spirulina laxissima* using 16S RNA sequence. The dry biomass, chlorophyll content, protein, phycocyanin, antimicrobial and antioxidant properties of the isolated strains were also analyzed. The strain proved to be a potential value-added product by showing rich protein, chlorophyll, high antimicrobial and antioxidant properties. The cytotoxicity analysis proved to be nontoxic and therefore the value-added products such as *Spirulina* cake, chocolate, tea, vermicelli and juice were made for vulnerable population due to its high nutritive value. This is the first study to report the preparation of value-added products from a freshwater algae *Spirulina laxissima*. In addition, there is a paucity of literature determining the biomass, chlorophyll, phycocyanin, antimicrobial and antioxidant contents of *Spirulina laxissima.* Hence, our research has unleased the great potentialities of *Spirulina laxissima.* It is suggested that the value-added product from this freshwater algae is considered organic and has significant health benefits over high-value chemical products. 

## Data Availability

Data will be made available upon reasonable request to corresponding author.
